# DDP-FedFV: A Dual-Decoupling Personalized Federated Learning Framework for Finger Vein Recognition

**DOI:** 10.3390/s24154779

**Published:** 2024-07-23

**Authors:** Zijie Guo, Jian Guo, Yanan Huang, Yibo Zhang, Hengyi Ren

**Affiliations:** 1 School of Computer Science, Nanjing University of Posts and Telecommunications, Nanjing 210023, China; b21030211@njupt.edu.cn (Z.G.); b21030229@njupt.edu.cn (Y.Z.); 2 Jiangsu High Technology Research Key Laboratory for Wireless Sensor Networks, Nanjing University of Posts and Telecommunications, Nanjing 210023, China; b21041607@njupt.edu.cn; 3 College of Automation & College of Artificial Intelligence, Nanjing University of Posts and Telecommunications, Nanjing 210023, China; 4 College of Information Science and Technology and College of Artificial Intelligence, Nanjing Forestry University, Nanjing 210037, China; renhy@njfu.edu.cn

**Keywords:** finger vein recognition, personalized federated learning, dual decoupling, two-phase training

## Abstract

Finger vein recognition methods, as emerging biometric technologies, have attracted increasing attention in identity verification due to their high accuracy and live detection capabilities. However, as privacy protection awareness increases, traditional centralized finger vein recognition algorithms face privacy and security issues. Federated learning, a distributed training method that protects data privacy without sharing data across endpoints, is gradually being promoted and applied. Nevertheless, its performance is severely limited by heterogeneity among datasets. To address these issues, this paper proposes a dual-decoupling personalized federated learning framework for finger vein recognition (DDP-FedFV). The DDP-FedFV method combines generalization and personalization. In the first stage, the DDP-FedFV method implements a dual-decoupling mechanism involving model and feature decoupling to optimize feature representations and enhance the generalizability of the global model. In the second stage, the DDP-FedFV method implements a personalized weight aggregation method, federated personalization weight ratio reduction (FedPWRR), to optimize the parameter aggregation process based on data distribution information, thereby enhancing the personalization of the client models. To evaluate the performance of the DDP-FedFV method, theoretical analyses and experiments were conducted based on six public finger vein datasets. The experimental results indicate that the proposed algorithm outperforms centralized training models without increasing communication costs or privacy leakage risks.

## 1. Introduction

Finger vein recognition technology is an emerging biometric technology that uses the distribution pattern of veins within the finger for identity verification. This technology is highly accurate, has live detection capabilities, and is noninvasive, making it suitable for various identity verification applications.

To extract more representative finger vein features, deep learning algorithms have become the mainstream feature extraction methods in finger vein recognition, considerably improving recognition accuracy [1,2,3,4]. Training a robust deep learning model typically requires a large amount of finger vein image data [4,5]. However, due to privacy protection laws [6,7,8,9], research institutions and companies cannot collect or share extensive finger vein datasets. As a result, these organizations can train models using only limited local data, leading to suboptimal accuracy and generalizability. Federated learning (FL) addresses this issue by enabling multiple clients to collaboratively train a model while ensuring data privacy and security. FL methods facilitate the training of high-performance models without sharing datasets, aligning with the privacy and high-accuracy requirements in biometric recognition, and thus shows great application potential. Currently, FL methods have been applied in areas such as facial recognition [10,11,12,13,14] and iris recognition [15,16]. However, research on finger vein recognition remains limited. Only one published study has proposed FedFV [17]. However, in this study, only the size of the dataset for each client was used to calculate the aggregation weight matrix, while the heterogeneity among clients’ finger vein datasets was not considered, resulting in significantly different performance across clients.

**Figure 1:**
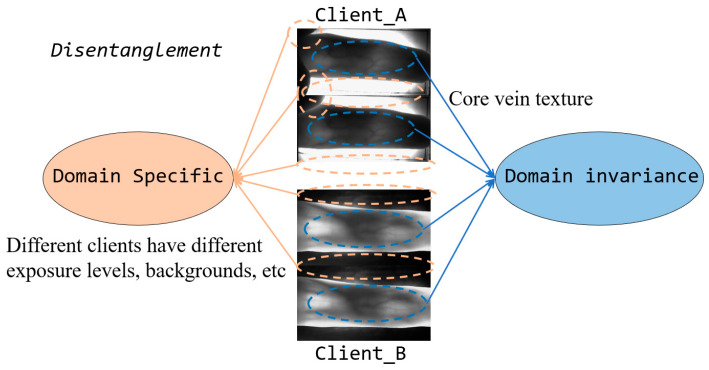
Example diagram illustrating the necessity of decoupling. Finger vein images can be divided into two parts: background and foreground information. In this paper, we refer to them as domain-specific and domain-invariant information, respectively. The former reflects the personalized information of specific datasets, such as exposure and imaging conditions for different clients. The latter refers to the core vein texture, which is common and is the key feature for finger vein recognition technology.

The heterogeneity among client datasets must be addressed when applying FL to finger vein recognition. In reality, researchers at institutions and companies collect finger vein images under different conditions with various equipment, resolutions, external environments, and capture areas, resulting in highly heterogeneous image sets. Existing methods have not effectively addressed this heterogeneity, leading to suboptimal performance. Figure 1 shows images from two different client datasets. Clearly, the main parts of these images are similar, consisting of vein patterns, but there are substantial differences in lighting, angle, background, and other aspects. In finger vein model training, the primary task is to accurately capture and analyze vein pattern features to construct a highly robust recognition model. However, during actual training, the model learns parameter information from the input images, which include both valuable foreground vein information and considerable background information. When model parameters, which couple various information, for different clients are uploaded to the server for aggregation, the global model parameters are influenced by the specific background information in the images of each client. This can reduce the generalizability of the aggregated model. A reasonable approach would be to distinguish foreground and background information. Foreground information, as common knowledge in finger vein recognition tasks, should be aggregated at the server. In contrast, background information, reflecting the personalized characteristics of each client, should remain local to mitigate the negative impact of dataset heterogeneity on model performance.

Based on the above analysis, this paper introduces the concept of decoupling [14] to better utilize both types of information. Decoupling is performed at two levels: the model level and the feature level. Model decoupling involves separating the feature extractor from the classifier, with the former focusing on improving model generalizability and the latter focusing on capturing personalized information. Additionally, feature decoupling is introduced to distinguish between foreground and background information. The decoupled common foreground information is used for collaborative training to obtain a global domain-invariant model with strong generalizability. The personalized background information is used in the second stage for client similarity measurements to train personalized models for each client. Based on the above ideas, this paper proposes the two-stage dual-decoupling personalized federated learning framework for finger vein recognition (DDP-FedFV), which protects data privacy while training high-performance personalized models for each client. The main contributions of this paper can be summarized as follows.

(1) In this paper, the concepts of generalizability and personalization are combined by designing a two-stage FL algorithm. In the first stage, the generalizability of the global server model is improved, while in the second stage, personalized information is emphasized during fine-tuning to derive model parameters better suited for each client (N-Model Personalized FL).

(2) To enhance model generalizability, this paper introduces a dual-decoupling strategy in the first stage of training. This is the first work in the field of finger vein recognition to incorporate this approach. The server aggregates the common information from various heterogeneous datasets to train a more robust model.

(3) To obtain personalized models for each client, an innovative personalized federated aggregation scheme, FedPWRR, is proposed in this framework. FedPWRR fully leverages the similarity between clients and the size of the datasets to calculate personalized aggregation weights. This weighted aggregation strategy enables the development of high-performance personalized models for each client.

(4) This paper presents a theoretical analysis of the convergence of the DDP-FedFV method. Experiments were conducted based on six public heterogeneous finger vein datasets, with the results demonstrating that the DDP-FedFV algorithm performs stably and reliably for clients with significantly different data distributions.

## 2. Related Work

### 2.1. Finger Vein Recognition

There has been significant progress in finger vein recognition technology in recent years. The main processes include preprocessing, finger vein feature extraction, and finger vein feature matching. Feature extraction is the core step, and two main types of methods have been developed: traditional methods and deep learning-based methods.

Traditional finger vein feature extraction methods can be divided into four categories: minutia-based, dimensionality reduction-based, local texture-based, and vein pattern-based methods [18,19,20,21]. These methods can achieve a certain level of accuracy. However, because they rely on manually designed features, they are highly subjective and may require redesigning features or adjusting parameters when handling new datasets, resulting in poor scalability and robustness. In contrast, deep learning-based methods learn more comprehensive features without manual intervention. Thus, deep learning-based finger vein recognition methods have gradually replaced traditional methods and become popular research topics. Liu et al. [22] employed a seven-layer convolutional neural network (CNN) with five convolutional layers and two fully connected layers to process finger vein images. Chen et al. [23] proposed a finger vein recognition algorithm based on feature block fusion and a CNN, improving the deep network input by using a set of feature points in the finger vein images. Abbas [24] introduced a new hybrid architecture combining a CNN and long short-term memory (LSTM) network to enhance vein detection performance. Ma et al. [25] used an ant colony algorithm for region of interest (ROI)-extraction and integrated a dual attention network (DANet) with EfficientNetV2, providing new ideas for deep learning-based finger vein recognition methods.

Overall, deep learning methods outperform traditional methods that require manual feature extraction. However, deep learning methods typically require large amounts of data for model training. Due to privacy protection, data security, and cost constraints, it is challenging for individual research institutions to collect large amounts of finger vein data, and they cannot share their datasets. Consequently, the lack of sufficient data is increasingly becoming a bottleneck for enhancing network performance. In this context, enabling multiple institutions to share data to collaboratively train more effective models while meeting privacy requirements has become increasingly important.

### 2.2. Finger Vein Recognition Based on Federated Learning

As a distributed machine learning framework, FL [26] enables model training through collaboration among clients without sharing raw data. FL methods simultaneously protect the privacy of client data and enhance model performance for each client, making FL highly suitable for development requirements in the biometric recognition field.

Currently, FL has demonstrated its unique value and broad application potential in biometric recognition, with substantial progress in face recognition [10,11,12,13,14] and iris recognition [15,16]. However, the application of FL with finger vein recognition technology is still in the exploratory stage. Among the limited research, Lian et al. [17] were the first to propose a FL-based finger vein recognition algorithm, FedFV. They designed a personalized parameter aggregation method to address the issues of existing personalized aggregation algorithms, including long processing times and high computational costs. This method also mitigates the limitations of traditional FL algorithms, with clients with less data contributing less to the model. However, due to the strong heterogeneity of finger vein datasets, FedFV does not provide an optimal solution, resulting in limited model performance and poorer performance for some clients.

To address the aforementioned limitations, in this paper, generalizability and personalization are both considered to design a two-stage FL model. In the first stage, a dual-decoupling mechanism is utilized to enhance the generalizability of the global server model, mitigating the adverse effects of heterogeneity among different datasets. In the second stage, the FedPWRR algorithm is designed to use the similarity information of the decoupled background features from the first stage to customize the model parameters for each client.

## 3. Methodology

This section provides a detailed introduction to the problem definition of the FL-based finger vein recognition algorithm. Building on this, the overall framework of the DDP-FedFV method and the detailed design of each module are introduced.

### 3.1. Problem Description

This section defines the problem of the FL-based finger vein recognition algorithm. Assume there are *N* different clients in the client set ***C***, denoted as ***C**_k_*(*k* = 1, ……, *N*). For each *k*-th client, there is a local private dataset *D_k_*∈*X_k_* × *Y_k_*, where *X_k_* and *Y_k_* represent the feature space and label space of the *k*-th client’s dataset, respectively. The size of each private dataset is *M_k_*, and the total size of all the clients’ datasets is *M*. In addition to the *N* clients, a server is defined to perform operations such as model parameter initialization and aggregation during each round of communication. After the global (*t* − 1)-th round of communication, the server aggregates the model parameters *θ^t^* and distributes them to each client. The objective definition of the FL-based finger vein recognition algorithm is shown in Equation (1).

(1) $$\underset{\theta }{\min}R_{emp}\left(\theta \right)=\frac{1}{N}\underset{k=1}{\overset{N}{\Sigma }}f_{k}\left(\theta \right)$$

Here, $R_{emp}\left(\theta \right)$ represents the empirical risk of the model parameters *θ* across all participating clients. $f_{k}\left(\theta \right)=E_{\left(x,y\right)\sim D_{k}}\left[L\left(\theta ;\left(x,y\right)\right)\right]$ denotes the empirical risk of the model parameters *θ* for the *k*-th client. $L\left(\theta ;\left(x,y\right)\right)$ is the loss function defined by the algorithm, where *x* and *y* represent the features and labels of the samples in dataset *D_k_*.

### 3.2. Framework of the DDP-FedFV Method 

**Figure 2:**
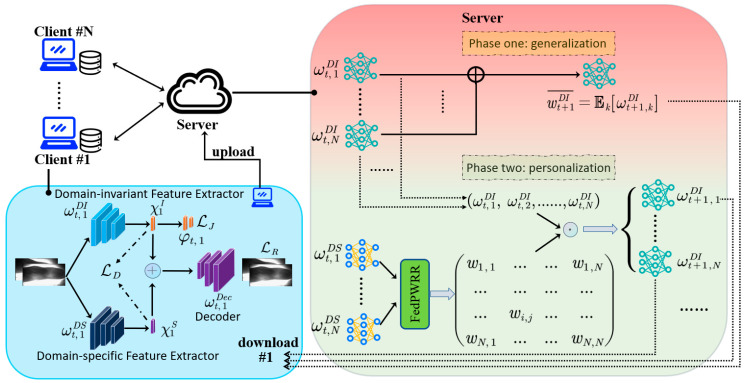
DDP-FedFV framework for finger vein recognition. The training process of this framework is divided into two main stages. In the first stage (generalization phase), the model is decoupled into two parts: a feature extractor and a classifier. To further decouple the dataset into foreground and background information, two feature extractors are devised for each client: domain-invariant and domain-specific extractors. This decouples the local dataset’s feature representations into domain-invariant and domain-specific parts. During this phase, the parameters of the domain-invariant model for each client are uploaded to the server, allowing the training of a model with strong generalizability. In the second stage (personalization phase), the server uses the size and distribution similarity information of each client’s dataset to determine the aggregation weight matrix W, customizing the model for each client to enhance personalization.

This section introduces the framework of the DDP-FedFV method. A schematic diagram is shown in Figure 2. The overall training process is divided into two stages. In the first stage, the heterogeneity among finger vein datasets is addressed by applying a dual-decoupling strategy to the local models. In the second stage, a similarity-based aggregation scheme is used to personalize and fine-tune the performance of the global model obtained in the first stage.

The pseudocode for the DDP-FedFV method is shown in Algorithm 1. For more detailed descriptions of these two stages, see Section 3.3 and Section 3.4. We define the total number of global communication rounds for the entire framework as *T*. The proportion of global communication rounds allocated to the first stage is *ρ*, meaning that the number of global communication rounds in the first stage *Tg* is *ρT*. Consequently, the number of global communication rounds in the second stage is *T-Tg*.
**Algorithm 1.** DDP-FedFV$\mathrm{Parameters}:\;\mathrm{Number}\;\mathrm{of}\;\mathrm{global}\;\mathrm{epochs}\;T,\;\mathrm{number}\;\mathrm{of}\;\mathrm{local}\;\mathrm{epochs}\;E,\;\mathrm{weight}\;\mathrm{aggregation}\;\mathrm{matrix}\;W\in S^{N\times N}$, generalization ratio
ρ $\mathrm{Initialize}\;\omega^{DI}$$,\;\omega^{DS}$$,\;\omega^{Dec},$φ $\mathrm{Compute}\;T_{g}\leftarrow \left\lfloor \rho T\right\rfloor ;T_{P}\leftarrow T-T_{g}$**Generalization Phase** Server executes:  **for** *t* = 0, 1, ……, (*T_g_* − 1) **do**    **for** each client *k* parallel **do**      $\left(\omega_{t,k}^{DI},\omega_{t,k}^{DS},\omega_{t,k}^{Dec},\varphi_{t,k}\right)\leftarrow \mathrm{ClientUpdate}\left(k,\omega_{t,k}^{DI},\omega_{t,k}^{DS},\omega_{t,k}^{Dec},\varphi_{t,k}\right)$      $\omega_{t,k}^{DS},\omega_{t,k}^{Dec},\varphi_{t,k}$ always stored locally without communication.     
$\mathrm{Obtain}\;\mathrm{the}\;\omega_{t}^{DI}$
 for this round ***t*** through Equation (6) and send it to all clients.**Personalization Phase** Server executes:  Use FedPWRR to compute the weight matrix *W*  **for** *t* **=** *T_g_*, ……, *T* **do**    **for** each client *k* parallel **do**      $\left(\omega_{t,k}^{DI},\omega_{t,k}^{DS},\omega_{t,k}^{Dec},\varphi_{t,k}\right)\leftarrow \mathrm{ClientUpdate}\left(k,\omega_{t,k}^{DI},\omega_{t,k}^{DS},\omega_{t,k}^{Dec},\varphi_{t,k}\right)$      $\omega_{t,k}^{DS},\omega_{t,k}^{Dec},\varphi_{t,k}$ always stored locally without communication.    **for** each client *k* parallel **do**      $\mathrm{Obtain}\;\mathrm{the}\;\omega_{t+1,k}^{DI}$ through Equation (8)      $\mathrm{Send}\;\omega_{t+1,k}^{DI}$ to client ***k*** and simultaneously update the parameters. $\mathrm{ClientUpdate}(k,\;\omega_{t}^{DI},$
$\omega_{t}^{DS},$
$\varphi_{t},$
$\omega_{t}^{Dec}$):    **/**/run for Client ***k***  $\mathrm{Set}\;\omega_{t,k}^{DI}\leftarrow \omega_{t}^{DI}$$,\;\omega_{t,k}^{DS}\leftarrow \omega_{t}^{DS}$$,\;\varphi_{t,k}\leftarrow \varphi_{t}$$,\omega_{t,k}^{Dec}\leftarrow \omega_{t}^{Dec}$  $L\left(\omega_{k}^{DI},\omega_{k}^{DS},\omega_{k}^{Dec},\varphi_{k}\right)=\mathrm{Equation}\left(2\right)$  **for** each local epoch **do**    $\left(\omega_{t+1,k}^{DI},\omega_{t+1,k}^{DS},\omega_{t?,k}^{Dec},\varphi_{t?,k}\right)=\left(\omega_{t,k}^{DI},\omega_{t,k}^{DS},\omega_{t,k}^{Dec},\varphi_{t,k}\right)-\eta \nabla L\left(\omega_{t,k}^{DI},\omega_{t,k}^{DS},\omega_{t,k}^{Dec},\varphi_{t,k}\right)$  $\mathrm{return}\;\left(\omega_{t+1,k}^{DI},\omega_{t+1,k}^{DS},\omega_{t+1,k}^{Dec},\varphi_{t+1,k}\right)$


### 3.3. The First Phase of the DDP-FedFV Method 

The primary function of this phase is to train a global model with strong generalizability. The core idea is to use a dual-decoupling mechanism, namely, model decoupling and feature decoupling. This section provides a detailed explanation of these mechanisms. Additionally, specific details of the FL process, such as the configuration of the loss function and the parameter aggregation method executed on the server, are thoroughly discussed.

The first decoupling performed for each client is model decoupling, which involves separating the neural network into two parts: the feature extractor and the classifier. In traditional FL frameworks, the model parameters for all clients are uploaded to the server for aggregation. However, this method is not suitable for practical finger vein recognition applications because the number of finger vein categories varies across clients, leading to different dimensions in the classifiers of each client. This discrepancy directly hinders parameter aggregation. Therefore, in the DDP-FedFV method, the model parameters are decoupled into two parts, $\theta_{k}=\omega_{k\;\;}\circ \varphi_{k}$, where $\omega_{k}$ is the feature extractor of the *k*-th client and $\varphi_{k}$ is the classifier of the *k*-th client. During the FL process, the feature extractor parameters for each client, which have the same dimensions, are uploaded for aggregation. This helps to improve model generalizability. However, the classifier parameters of each client remain local because they effectively capture the distribution patterns of the local data [27,28], thereby benefiting the personalization of the client models.

Next, the second decoupling of the model—feature decoupling—is performed. This involves extracting domain-invariant and domain-specific features from finger vein images using two corresponding models. After the first decoupling, the trained feature extractor parameters still contain considerable domain-specific information related to the background information and image noise in the finger vein images. These features vary significantly among the finger vein datasets of different clients. Directly aggregating these coupled feature extractor parameters is not conducive to improving model generalizability. Inspired by [14], this paper introduces domain disentanglement learning. The feature extractor of the *k*-th client (*k* = 1,2,…,*N*) is divided into two parts: domain-invariant $\omega_{k}^{DI}$and domain-specific $\omega_{k}^{DS}$. This approach decouples the feature representations in the client’s dataset into domain-invariant and domain-specific features. The domain-invariant model parameters are uploaded to the server to train a global model with strong generalizability. Moreover, both the classifier *φ* and domain-specific model parameters are kept local to capture the distribution characteristics of the local dataset. These can be used in the second stage for client similarity measurements.

Next, we provide a detailed introduction to the training process, including the choice of the backbone network, the loss function configuration, and the server parameter aggregation method used in this phase. We adopted MobileNetV3 [29] as the backbone network for the feature extractor. The loss function for the *k*-th finger vein client is designed as shown in Equation (2).

(2) $$L\left(\omega_{k}^{DI},\omega_{k}^{DS},\omega_{k}^{Dec},\varphi_{k}\right)=L_{J}\left(\omega_{k}^{DI},\varphi_{k}\right)+L_{D}\left(\omega_{k}^{DI},\omega_{k}^{DS}\right)+L_{R}\left(\omega_{k}^{DI},\omega_{k}^{DS},\omega_{k}^{Dec}\right)$$

The combined loss function $L_{J}$ is used to obtain the classification loss, as calculated in Equation (3). In this equation, $L_{S}$ represents SoftmaxLoss [30], $L_{C}$ represents CenterLoss [31], and λ is a hyperparameter used to balance the weights of SoftmaxLoss and CenterLoss. During the training of the local model of the *k*-th client, the domain-invariant feature $\chi_{k}^{I}$ is fed into the classifier $\varphi_{k}$ to obtain the classification loss.

(3) $$L_{J}\left(\omega_{k}^{DI},\varphi_{k}\right)=L_{S}+\lambda L_{C}$$

$L_{D}$is the loss, which is calculated as shown in Equation (4). To ensure that features $\omega_{k}^{DI}$and $\omega_{k}^{DS}$adequately represent the domain-invariant and domain-specific information of the finger vein data, the soft subspace orthogonality constraint [14] is applied in Equation (4).

(4) $$L_{D}\left(\omega_{k}^{DI},\omega_{k}^{DS}\right)=\underset{x\sim D_{i}}{\Sigma }||{\left(\chi_{k}^{I}\right)}^{T}\left(\chi_{k}^{S}\right)|{|}_{F}^{2}$$

$L_{R}$ is the reconstruction loss, which is calculated as shown in Equation (5). The domain-invariant feature $\chi_{k}^{I}$ and domain-specific feature $\chi_{k}^{S}$ extracted for the *k*-th client are input into a decoder to reconstruct an image consistent with the original finger vein image. The difference between the generated image and the actual input image during this process results in reconstruction errors. This loss function is optimized to enhance the model’s ability to capture important information from the original data, allowing the model to learn high-quality parameters. Here, $\omega_{t,k}^{Dec}$ is the local image decoder of the *k*-th client in the global *t*-th communication round. This decoder uses domain-invariant and domain-specific features to reconstruct the finger vein image, which is used to calculate the reconstruction loss.

(5) $$L_{R}\left(\omega_{k}^{DI},\omega_{k}^{DS},\omega_{k}^{Dec}\right)=\underset{x\sim D_{i}}{\Sigma }||Dec_{k}\left(\chi_{k}^{I}+\chi_{k}^{S}\right)-x|{|}_{2}^{2}$$

In the first stage, the domain-invariant models of each client are aggregated on the server using the method described in Equation (6).

(6) $$\bar{w_{t+1}^{DI}}=E_{k}[\omega_{t+1,k}^{\mathrm{DI}}]$$

Here, $\bar{w_{t+1}^{DI}}$ refers to the expected value obtained by the server after aggregating the domain-invariant model parameters from all clients in the *t*-th global communication round. The server then distributes this aggregated value to each client.

### 3.4. The Second Phase of the DDP-FedFV Method 

This section primarily introduces the core algorithm used in the second stage, FedPWRR, and the FL process applied during this stage. The main objectives in this phase are to measure the similarity of the data distributions across clients and to obtain personalized aggregated model parameters for each client, which further improves model performance.

The main objective of FedPWRR is to utilize the similarity of the data distributions across clients to generate a weight matrix. In the server, this matrix is used to customize the model parameters for each client. The similarity of the data distributions is measured based on domain-specific model information. After the first stage of training, the domain-specific model captures information that reflects the distribution of the finger vein datasets. The similarity between domain-specific models is used to obtain the similarity matrix between clients. FedPWRR calculates the weight aggregation matrix based on this similarity matrix and the size of each client’s dataset to personalize the domain-invariant model parameters aggregated for each client.

To describe the FedPWRR algorithm, the *trans* function is defined, as shown in Equation (7).

(7) $$trans\left(q_{1},q_{2},\ldots \ldots ,q_{N}\right)=\left(\frac{q_{1}}{\Sigma_{i=1}^{N}q_{i}},\frac{q_{2}}{\Sigma_{i=2}^{N}q_{i}},\ldots \ldots ,\frac{q_{N}}{\Sigma_{i=N}^{N}q_{N}}\right)$$

The input to the *trans* function is a one-dimensional vector $\left(q_{1},q_{2},\ldots \ldots ,q_{N}\right)$, and the output is also a one-dimensional vector.

The pseudocode for the second stage of the algorithm, FedPWRR, is shown in Algorithm 2.
**Algorithm 2.** FedPWRR$\mathrm{Parameters}:\;\mathrm{Size}\;\mathrm{of}\;\mathrm{each}\;\mathrm{client}\;\mathrm{dataset}\;M_{k}(k=1,2,\ldots \ldots ,N),\;\mathrm{domain}\text{-}\mathrm{specific}\;\mathrm{feature}\;\mathrm{extractor}\;\mathrm{for}\;\mathrm{each}\;\mathrm{finger}\;\mathrm{vein}\;\mathrm{client}\;\omega_{T_{g},k}^{DS}$, weights assigned to clients with negative similarity ***r***, base weight scaling factor for each client *rr* **Initialize** Two empty matrices *s* and *W*_0_ to store the results of intermediate calculations**Server executes:**  Step 1: Obtain (*e*_1_, *e*_2_, ……, *e_N_*) via the trans operation based on (*M*_1_, *M*_2_, ……, *M_N_*).  $\mathrm{Step}\;2:\;\mathrm{The}\;\mathrm{similarity}\;\mathrm{assessment}\;\mathrm{is}\;\mathrm{performed}\;\mathrm{based}\;\mathrm{on}\;\mathrm{the}\;\mathrm{parameters}\;\mathrm{of}\;\mathrm{the}\;\mathrm{last}\;\mathrm{layer}\;\mathrm{of}\;\omega_{T_{g},k}^{DS}$ uploaded for each finger vein client, and a symmetric similarity matrix Φ is obtained using the cosine similarity assessment algorithm.  Step 3:  **for** each client *k* parallel **do**    The total number of negative clients is counted as **cnt**.    **for**
*i*-th client with **negative** similarity with client *k*
**do**      $s[k][i]=\frac{r}{\max(cnt,\;1)}$    **for**
*j*-th client with **positive** similarity with client *k*
**do**      $s\left[k\right]\left[j\right]=\frac{\Phi \left[k\right]\left[j\right]}{\Sigma \Phi \left[k\right]\left[u\right]}\times \left(1-r\right),where\;\Phi \left[k\right]\left[u\right]>0$    set *s*[*k*][*k*] = 1 and then let *s*[*k*] = trans(*s*[*k*])    Then, *W*_0_[*k*] = trans((*e*_1_, *e*_2_, ……, *e_N_*)○*s*[*k*])  Step 4: The individual weights of the final personalized aggregation matrix can be calculated using the following equation:$\;\;\;\;\;W\left[k\right]\left[j\right]=\left\{\begin{array}{l} \left(1-rr\right)+rr\times W_{0}\left[k\right]\left[u\right],when\;k=u\\ rr\times W_{0}\left[k\right]\left[u\right],other \end{array}\right.$  Return the weight matrix *W*

Through the above steps, the server can compute the aggregation weight matrix *W* at the beginning of the second stage, which is then used in the subsequent personalized aggregation process. In the second stage, FedPWRR needs to calculate the aggregation weight matrix only once, resulting in low computational overhead. Moreover, this approach comprehensively considers the similarity of data distributions across clients, making more efficient use of the available information.

The following section describes how the server aggregates personalized model parameters for each client during the second stage of training. The server uses the aggregation weight matrix *W* calculated by FedPWRR to update the domain-invariant model parameters for the *k*-th client according to Equation (8).

(8) $$\omega_{t+1,k}^{DI}=\underset{j\in \mathbb{C}}{\Sigma }\left(W\left[k\right]\left[z\right]\times \omega_{t,z}^{DI}\right)$$

where $\omega_{t+1,k}^{DI}$ represents the domain-invariant feature extractor parameters of the *k*-th client in the (*t* + 1)-th global communication round; $\omega_{t,z}^{DI}$ represents the domain-invariant feature extractor parameters of the *z*-th client in the *t*-th global communication round; *W* is the aggregation weight matrix calculated by the FedPWRR algorithm; and ℂ represents the set of all finger vein clients.

## 4. Convergence Analysis

This section provides a proof of the convergence of the proposed algorithm. The algorithm consists of two stages, with the first generalization stage being the key component of the proposed algorithm. Therefore, the convergence proof focuses primarily on the first stage, and demonstrating the convergence of the first stage implies the overall convergence of the algorithm. Additionally, this section proves that the DDP-FedFV algorithm maintains good convergence properties even when some clients do not participate. The relevant assumptions and definitions for the proof are as follows.

- μ strongly convex of *L_k_*

(9) $$\left\|\nabla L_{k}\left(w_{1}\right)-\nabla L_{k}\left(w_{2}\right)\|\geq \mu \|w_{1}-w_{2}\right\|$$

- Jensen inequality

If *f* is a convex function defined on a real interval *I* and *X* is a random variable taking values in *I*, then the Jensen inequality can be stated as:

(10) $$f\left(E\left[X\right]\right)\leq E\left[f\left(X\right)\right]$$

where *E*[*X*] represents the expected value of the random variable *X*. This inequality is frequently used in the proof.

- γ−inexact solution [32]

For the local client function $h_{k}\left(w_{k}^{DI},w_{k}^{DS},w_{k}^{Dec},\varphi_{k};w_{k}^{DI}\right)=L_{k}\left(w_{k}^{DI},w_{k}^{DS},w_{k}^{Dec},\varphi_{k}\right)$ with $\gamma \in \left[0,1\right]$, $w_{k,*}^{DI}$ is defined as the γ-inexact solution of

$$\min_{w_{k}^{DI}}\left\{h_{k}\left(w_{k}^{DI},w_{t+1,k}^{DS},w_{t+1,k}^{Dec},\varphi_{t+1,k}\right)\right\}$$

if:

(11) $$\left\|\nabla h_{k}\left(w_{k,*}^{DI},w_{t+1,k}^{DS},w_{t+1,k}^{Dec},\varphi_{t+1,k}\right)\|\leq \gamma \|\nabla h_{k}\left(w_{t,k}^{DI},w_{t+1,k}^{DS},w_{t+1,k}^{Dec},\varphi_{t+1,k}\right)\right\|$$

- B-local dissimilarity [32]

The local function *L_k_* is B-locally dissimilar at $w_{t}^{DI}$ if:

(12) $$E_{k}\left[\left\|\nabla L_{k}\left(w_{t,k}^{DI},w_{t,k}^{DS},w_{t,k}^{Dec},\varphi_{t,k}\right)\right\|\right]\leq \|\nabla f\left(w_{t}^{DI}\right){\|}^{2}B^{2}$$

and B is defined as:

(13) $$B\left(w_{t}^{DI}\right)=\sqrt{\frac{E\left[∥\nabla L_{k}\left(w_{t,k}^{DI},w_{t,k}^{DS},w_{t,k}^{Dec},\varphi_{t,k}\right)∥\right]}{∥\nabla f\left(w_{t}^{DI}\right){∥}^{2}}}$$

Based on the above assumptions and definitions, we can derive the convergence result of the algorithm.

**Theorem 1.**  *If the chosen learning rate η, the total number of participating clients K, γ, and μ satisfy*

(14) $$\alpha =\left(\frac{\eta }{2}\left(B^{2}+1\right)-\frac{BL(1+\gamma )}{2\mu }-(2\sqrt{2}+2)\frac{B^{2}L{\left(1+\gamma \right)}^{2}}{\mu K}-\frac{\sqrt{2}B(1+\gamma )}{\mu \sqrt{K}}\right)>0$$

Then, according to the global objective at round *t*, the expected decrease is expressed as shown in Equation (15).

(15) $$E_{St}\left[f\left(w_{t+1}^{DI}\right)\right]\leq f\left(w_{t}^{DI}\right)-\alpha \|\nabla f\left(w_{t}^{DI}\right){\|}^{2}$$

where *St* is the set of clients selected in round *t*. The corresponding convergence form is then derived as follows:

(16) $$\frac{1}{T}\overset{T-1}{\underset{t=0}{\Sigma }}\|\nabla f\left(w_{t}^{DI}\right){\|}^{2}\leq \frac{f\left(w_{0}^{DI}\right)-f^{*}}{\alpha T}$$

where $f^{*}$ is the minimum value of the global model and $w_{0}^{DI}$ is the initial model parameters.

The detailed proof process is provided in Appendix A. The proof results demonstrate that the convergence of the DDP-FedFV framework is theoretically guaranteed. Additionally, in our proof, we allow some clients to perform only a few local updates or incomplete local training while still participating in global model aggregation. This setting is closer to real-world applications.

## 5. Experiments

This section describes the specific dataset information, testing methods, and evaluation metrics. In addition, the results of each experiment are analyzed.

### 5.1. Datasets and Verification Method

In this paper, to simulate real-world finger vein recognition applications, six public datasets are used for FL: SDUMLA-HMT [33], MMCBNU-6000 [31], HKPU-FV [21], NUPT-FV [34], VERA [35], and UTFVP [36]. The SDUMLA-HMT dataset contains 636 classes, with 6 photos per class; the MMCBNU-6000 dataset contains 600 classes, with 10 photos per class; the HKPU-FV dataset contains 600 classes, with 6 photos per class; the NUPT-FV dataset contains 1680 classes, with 10 photos per class; the VERA dataset contains 220 classes, with 2 photos per class; and the UTFVP dataset contains 360 classes, with 4 photos per class.

These six datasets are treated as different clients. Table 1 provides a brief introduction to these six datasets, including specific experimental settings. These finger vein datasets have different data distributions due to factors such as various acquisition devices and background noise, which exemplify the classic non-iid problem in FL. Mu et al. [37] explored the heterogeneity characteristics of finger vein datasets. NUPT, as the largest finger vein dataset currently available, shows poor performance in federated learning with other datasets due to different finger placement orientations. In contrast, HKPU, SDUMLA, and UTFVP exhibit similar finger placement positions, image background lighting, and other characteristics, resulting in good compatibility. VERA, being the smallest dataset in terms of data size, negatively impacts the performance of models trained in cooperation with other datasets. 

In the experiments, the open-set testing method, which is commonly employed in biometrics, was used. This method accurately reflects the model’s robustness and accuracy in real finger vein recognition scenarios and demonstrates the generalizability of the finger vein models better than other approaches. The specific datasets are divided as follows: each client’s finger vein dataset is randomly split into training and testing sets at an 8:2 ratio.

In the training phase, only the training set is used for model training. Once the model is trained, during the testing phase, verification matching pairs for each client are generated from the dataset images. Similarity measurements are performed based on the feature vectors extracted by the model. The image matching rule is as follows: if the similarity is greater than or equal to the preset threshold and the two images come from the same class, the match is considered correct; if the similarity is less than the preset threshold and the two images come from different classes, the match is also considered correct. All other cases are considered incorrect.

### 5.2. Evaluation Metrics

This subsection introduces the evaluation metrics used in the experiments, namely, the equal error rate (*EER*) and *TAR@FAR =* 0.01 [17,38,39,40], to comprehensively assess the performance of the proposed framework. Additionally, to provide a more intuitive comparison of algorithm performance, three statistical metrics were used.

In finger vein recognition, the *EER* is a widely used evaluation metric. The *EER* is the value at which the False Accept Rate (*FAR*) and False Reject Rate (*FRR*) are equal; the lower the value is, the better the model’s performance. The related *FAR* and *FRR* metrics are defined as shown in Equations (17) and (18).

(17) $$FAR=\frac{N_{FA}}{N_{IRA}}*100\%$$

(18) $$FRR=\frac{N_{FR}}{N_{GRA}}*100\%$$

where *N_FA_* is the number of false acceptances, *N_IRA_* is the number of interclass matching pairs, *N_FR_* is the number of false rejections, and *N_GRA_* is the number of intraclass matching pairs.

In addition, the *TAR* value when the *FAR* value is 0.01, abbreviated as *TAR@FAR =* 0.01, is a key metric in this experiment. Here, *TAR* is defined as *TAR =* 1 − *FRR*. The higher the value of *TAR@FAR =* 0.01 is, the better the model’s performance.

To more intuitively evaluate the algorithm’s performance, this paper also introduces three statistical metrics: best performance, worst performance, and average performance.

The best performance is defined as the best result achieved by the algorithm among all client metrics. This metric reflects the optimal performance the algorithm can obtain with different datasets and settings.

The worst performance is defined as the client with the poorest performance, which aids in evaluating the robustness and generalizability of the algorithm. In practical scenarios, finger vein data are distributed across different clients, each with distinct data distributions and characteristics. This metric allows us to better understand how the algorithm performs in complex environments with heterogeneous finger vein data, reflecting the model’s adaptability to the most challenging client data.

The average performance is a straightforward average metric that is well suited for measuring overall performance across all clients in FL. The use of this metric ensures that the performance of the model for each client is considered equally, regardless of the size of the corresponding client data, providing a fair and consistent evaluation standard that reflects the comprehensive effectiveness of the FL algorithm across different clients.

### 5.3. Experimental Results and Analysis

To evaluate the performance of the DDP-FedFV framework, four sets of experiments were conducted. In the first set of experiments, the DDP-FedFV method was compared with client-independent training and client-centralized training methods to validate the effectiveness of our framework. In the second set of experiments, the baseline results were compared with those of the first stage of the training method to verify the effectiveness of the first stage. In the third set of experiments, the performance using only the first stage was compared with that using the complete DDP-FedFV algorithm to test the effectiveness and importance of the second stage. In the fourth set of experiments, a comparative analysis with existing FL algorithms was conducted to validate the superiority of our proposed algorithm.

(1) Comparison with client-independent training and client-centralized training methods

In this experiment, “Local” represents client-independent training, where clients do not communicate with each other. “Centralized” indicates centralized training involving all clients. To better compare the performance of our algorithm with that of the other two algorithms, we set up two validation experiments: “Centralized_1” and “Centralized_2”. In both experiments, the model parameters from centralized training were used. The difference is that in the “Centralized_1” experiment, the metrics for each of the six clients were measured separately, while in the “Centralized_2” experiment, the six clients were combined into one large dataset and the overall metrics were measured. “DDP-FedFV” is the method proposed in this paper. In the *EER* column, smaller values indicate better performance. In the *TAR*@*FAR* = 0.01 column, higher values indicate superior performance.

The experimental results in Table 2 demonstrate that the “Centralized” training method performs considerably better than the “Local” training method. However, due to the heterogeneity of different finger vein data distributions, the performance of this model still has certain limitations. By using feature decoupling to obtain domain-invariant information for communication and employing a personalized aggregation mechanism in the second stage, the proposed method effectively overcomes the strong heterogeneity among different finger vein datasets. This further enhances the performance of the trained model. Therefore, the performance of the proposed DDP-FedFV framework is comparable or superior to that of the centralized training method.

**Figure 3:**
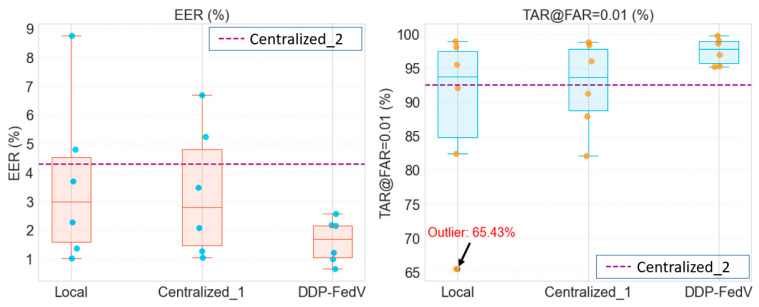
Boxplot of this experiment.

To better and more intuitively analyze the experimental results, we plotted boxplots of the above experimental results, as shown in Figure 3.

This paper will also statistically compare the performance of each client of the algorithm to identify outliers and mark them in a box plot. According to the *EER* results, the “Local” method obtains a wide distribution of values, with a median of approximately 3%, indicating relatively high variability and poor overall performance. The “Centralized_1” method has a lower median *EER* than the “Local” method but still shows high variability. The “DDP-FedFV” method obtains the lowest median *EER*, indicating the best performance among these methods, and the data are relatively concentrated, suggesting that the proposed method performs consistently across different clients. Next, the *TAR*@*FAR* = 0.01 results were analyzed. The “Local” method also performs the worst, with a significant outlier indicating much poorer performance with one client compared to with the other clients. The “Centralized_1” method performs better than the “Local” method, with a median exceeding 90% and relatively low variability, suggesting that it maintains high performance across multiple clients. The “DDP-FedFV” method shows outstanding performance in terms of the *TAR*@*FAR* = 0.01 metric, with a median close to 99%, and more tightly clustered data, further confirming the stability and superiority of the proposed algorithm across different clients. These results also demonstrate the necessity and effectiveness of introducing FL in finger vein recognition.

(2) Experiments to prove the validity of the first phase

The ablation experiments were primarily performed to validate the effectiveness of the designed two-stage approach. In this subsection, the performance of the first-stage method was compared with that of the baseline training method to verify the effectiveness of the first stage. FedPer [27], which uses a server aggregation method similar to FedAvg, was selected as the baseline. Additionally, to better demonstrate the feature decoupling in the first stage of our model, the Grad-CAM [41] method was employed to visualize the ROIs in the decoupled model. This comparison between our first-stage model and the baseline aims to demonstrate the effectiveness of the refined feature extraction approach applied in the first stage.

**Figure 4:**
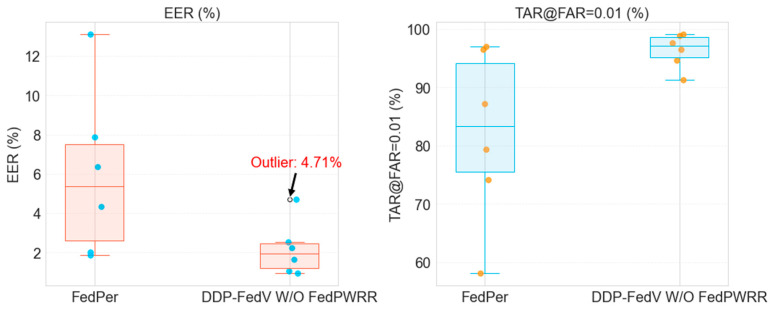
Boxplot depicting the experimental comparison with the baseline.

The results of the ablation experiments are shown in Table 3 and visualized as boxplots in Figure 4. Clearly, the two metrics of FedPer exhibit large interquartile ranges, indicating marked performance variability among clients, with performance based on the VERA dataset particularly lower than the average level. This is due to the substantial distribution differences among the six finger vein datasets, leading to serious “client drift” issues. In contrast, the metrics obtained from the first stage of our proposed algorithm have very small interquartile ranges, indicating that in the first stage, the algorithm shows highly consistent and reliable performance among different clients. Even in the worst cases, it maintains high recognition accuracy. The dual-decoupling design ensures fine-grained feature utilization, encourages the exchange of common information, and ensures good performance for all clients. Although the *EER* value of 4.71% for the VERA client is considered an outlier on the boxplot, this value is 64.076% less than the 13.111% *EER* of FedPer, and this value is optimized further in the second stage. Overall, the experiments confirm that the first stages use the domain-invariant model for global communication, resulting in a generalizable global model, which provides a solid foundation for ensuring good overall model performance. Next, we use neural network visualization methods to further validate the effectiveness of the feature-decoupling method applied in the first stage of the algorithm.

**Figure 5:**
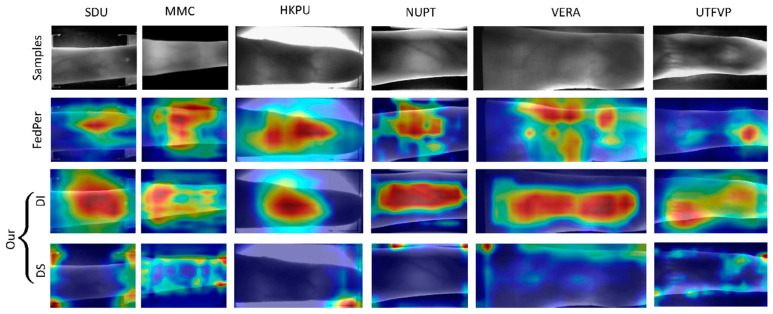
Grad-CAM visualization of the regions focused on by the domain-invariant (DI) and domain-specific (DS) models. In this subsection, the Grad-CAM [41] method is used to visualize the feature maps of the deep learning model, highlighting the areas of the image that the model focuses on most. The first row shows sample images for each client. The second row displays the heatmaps obtained with the baseline method selected in this paper. The third row presents heatmaps of the areas on which the domain-invariant (DI) model focuses. The fourth row shows heatmaps of the areas on which the domain-specific (DS) model focuses.

The model feature map visualizations are shown in Figure 5. As shown by the sample images for each finger vein dataset in the first row, there are appreciable differences in background information such as clarity, background color, and exposure, but the core vein region features are largely consistent among the different images. In the second row, the heatmaps obtained with the baseline training method show that the trained single model does not always capture the core region well. This is because the single model cannot adapt to the data distribution of each client, leading to deviations in the model’s focus areas from the core vein distribution regions for some clients.

In contrast, our method can finely separate the two types of image information. According to the domain-invariant (DI) model visualization results in the third row, the core finger vein region shows high brightness, indicating that the decoupled DI model effectively focuses on more general, generalized information. This type of information helps enhance the performance of the global model and is crucial for communication during FL. According to the domain-specific (DS) model visualization results in the fourth row, the model focuses more on areas outside the finger vein, such as the edges of the finger. These areas are unique to each client and should remain local without participating in global communication. The visualization results strongly validate the effectiveness of the dual-decoupling method applied in the first stage. This method is important for mitigating the heterogeneity among datasets.

(3) Experiments to prove the validity of the second phase

In this section, we compare the experimental results of models with and without the second stage, that is, with and without the personalized aggregation method *FedPWRR*, to verify the effectiveness and importance of the second stage.

The specific values of the aggregated weight matrices obtained in the second stage are detailed in Appendix B. Table 4 shows a comparison of the experimental results of the DDP-FedFV method without the second stage and those of the complete DDP-FedFV framework. Clearly, incorporating the second stage results in improvements across different clients. The average *EER* of the DDP-FedFV method was 2.563%, which is 25.902% less than that of the algorithm without the second stage. Moreover, the average *TAR*@*FAR* = 0.01 of the DDP-FedFV method is 95.15%, which is 1.163% higher than that of the model without the second stage, indicating that the second stage plays a crucial role in enhancing the overall performance. The worst *EER* of the DDP-FedFV method was 2.563%, which is 45.584% less than that of the algorithm without the second stage, and the worst *TAR*@*FAR* = 0.01 of the DDP-FedFV method was 95.15%, which is 4.285% higher than that of the model without the second stage. The models with the worst performance are typically associated with clients with data heterogeneity, and the second-stage design further mitigates the impact of this heterogeneity.

**Figure 6:**
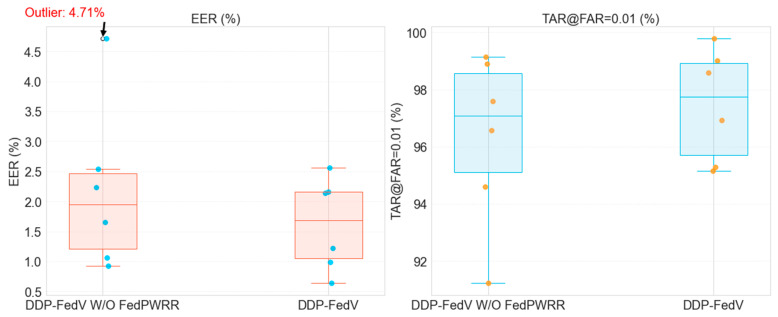
Boxplot of the ablation experiment.

Furthermore, the boxplot in Figure 6 reveals that the model trained without the *FedPWRR* method exhibits high variability, with the EER metric for the VERA client classified as an outlier. This shows that the method without the *FedPWRR* method has greater variability, with the models for some clients showing considerably poor performance. In contrast, the DDP-FedFV metric data are more concentrated and denser, and the median metrics are clearly improved compared to those of the algorithm without the second stage. This visually demonstrates the effectiveness of the proposed *FedPWRR* method and the rationality of the two-stage training paradigm.

(4) Experiments comparing the performance with those of existing algorithms

**Figure 7:**
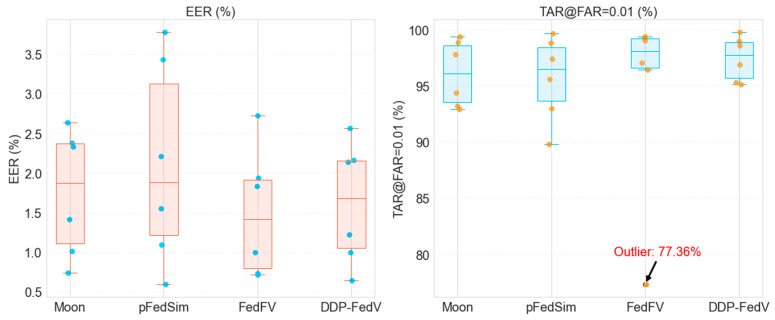
Boxplot of the experiment.

To our knowledge, the only FL-based finger vein recognition method is FedFV [17]. To comprehensively evaluate the superiority of the DDP-FedFV method, in addition to reproducing FedFV, we selected several mainstream FL algorithms for comparison, such as Moon [42], which combines FL with contrastive learning, and the personalized FL method pFedSim [43]. The analysis results in Table 5 and Figure 7 show that the DDP-FedFV method consistently obtains the optimal results for the best, worst, and average performance metrics. Notably, the model trained by our method ensures that even the worst-performing client model shows good performance. The average *EER* of the DDP-FedFV method was 1.622%, which was 7.473% and 23.237% lower than those of Moon and pFedSim, respectively, but 8.64% higher than that of FedFV (1.493%).

The average *TAR*@*FAR* = 0.01 of the DDP-FedFV method was 97.46%, which was 1.394%, 1.817%, and 2.838% greater than those of Moon, pFedSim, and FedFV, respectively, indicating an improvement in overall performance. The worst *EER* of the DDP-FedFV method was 2.563%, which was 2.769%, 32.178%, and 6.152% lower than that of Moon, pFedSim, and FedFV, respectively. The worst *TAR*@*FAR* = 0.01 of the DDP-FedFV method was 95.15%, which was 2.356%, 5.946%, and 22.996% greater than those of Moon, pFedSim, and FedFV, respectively. This demonstrates that our algorithm can maintain good performance even for clients with considerable heterogeneity, showing robust overall performance. Moreover, the boxplot in Figure 7 reveals that the models of each client obtained using our algorithm show good performance, with low overall variability and more stable performance. These results collectively prove the superiority of the DDP-FedFV method over existing algorithms.

## 6. Conclusions

This paper proposes a novel dual-stage FL framework, DDP-FedFV, which combines generalization and personalization. In the first stage, we focus on enhancing the generalizability of the global model, while in the second stage, we fine-tune the model parameters for each client. To improve the model’s generalizability, a dual-decoupling mechanism is applied in the first stage, and the parameters of the domain-invariant feature extractors for each client are uploaded for aggregation. To obtain customized high-performance models for each client, the FedPWRR algorithm is designed based on the first-stage decoupling algorithm. This algorithm uses client similarity information and dataset size to personalize the parameters aggregated for each client’s model. A theoretical analysis of the DDP-FedFV method shows the convergence of the algorithm. The experimental results demonstrate that the proposed DDP-FedFV framework outperforms existing methods, with superior results across multiple metrics.

## Figures and Tables

**Table 1:** Introduction to the datasets used in the experiment.

FV Datasets	Number of Fingers	Total Images	Training Set	Test Set	Number of Authentication Pairs
SDUMLA-HMT	636	3816	3054	976	289,941
MMCBNU-6000	600	6000	4800	1200	719,400
HKPU-FV	312	1872	1500	372	69,006
NUPT-FV	1680	16,800	13,440	3360	5,643,120
VERA	220	440	352	88	3828
UTFVP	360	1440	1152	288	41,328

**Table 2:** Comparison of local training, centralized training, and FL training.

Datasets	Local		Centralized_1		Centralized_2		DDP-FedFV	
	EER	TAR@FAR = 0.01	EER	TAR@FAR = 0.01	EER	TAR@FAR = 0.01	EER	TAR@FAR = 0.01
SDU	3.687%	92.05%	3.485%	91.27%	4.280%	92.47%	2.163%	96.92%
MMC	1.365%	98.13%	1.272%	98.43%			0.647%	99.78%
HKPU	2.265%	95.47%	2.072%	96.03%			1.225%	98.59%
NUPT	1.025%	98.93%	1.048%	98.86%			0.995%	99.02%
VERA	8.749%	65.43%	6.685%	82.07%			2.563%	95.29%
UTFVP	4.802%	82.45%	5.236%	87.90%			2.141%	95.15%
Best	1.025%	98.93%	1.048%	98.86%	4.280%	92.47%	0.647%	99.78%
Worst	8.749%	63.43%	6.685%	82.07%			2.563%	95.15%
Average	3.649%	88.74%	3.300%	92.43%			1.622%	97.46%

**Table 3:** Performance comparison between the baseline algorithm and the first-stage algorithm.

Datasets	FedPer		The First Phase Method	
	EER	TAR@FAR = 0.01	EER	TAR@FAR = 0.01
SDU	7.875%	79.41%	2.239%	96.58%
MMC	1.867%	96.49%	0.930%	99.14%
HKPU	4.348%	87.19%	1.654%	97.59%
NUPT	2.026%	97.06%	1.063%	98.90%
VERA	13.111%	58.05%	4.710%	91.24%
UTFVP	6.362%	74.17%	2.540%	94.61%
Best	1.867%	97.06%	0.930%	99.14%
Worst	13.111%	58.05%	4.710%	91.24%
Average	5.931%	82.06%	2.189%	96.34%

**Table 4:** Comparison between the performance of the first-stage algorithm and the complete algorithm.

Datasets	DDP-FedFV W/O FedPWRR		DDP-FedFV	
	EER	TAR@FAR = 0.01	EER	TAR@FAR = 0.01
SDU	2.239%	96.58%	2.163%	96.92%
MMC	0.930%	99.14%	0.647%	99.78%
HKPU	1.654%	97.59%	1.225%	98.59%
NUPT	1.063%	98.90%	0.995%	99.02%
VERA	4.710%	91.24%	2.563%	95.29%
UTFVP	2.540%	94.61%	2.141%	95.15%
Best	0.930%	99.14%	0.647%	99.78%
Worst	4.710%	91.24%	2.563%	95.15%
Average	2.189%	96.34%	1.622%	97.46%

**Table 5:** Comparison of the performance of the proposed method with those of existing finger vein recognition algorithms based on FL.

Datasets	Moon [42]		pFedSim [43]		FedFV [17]		DDP-FedFV	
	EER	TAR@FAR = 0.01	EER	TAR@FAR = 0.01	EER	TAR@FAR = 0.01	EER	TAR@FAR = 0.01
SDU	2.383%	94.43%	2.211%	95.61%	1.938%	97.10%	2.163%	96.92%
MMC	1.013%	98.90%	0.596%	99.68%	0.719%	99.41%	0.647%	99.78%
HKPU	1.416%	97.80%	1.557%	97.40%	0.736%	99.26%	1.225%	98.59%
NUPT	0.740%	99.39%	1.098%	98.80%	0.995%	99.03%	0.995%	99.02%
VERA	2.330%	93.24%	3.779%	89.81%	2.731%	77.36%	2.563%	95.29%
UTFVP	2.636%	92.96%	3.438%	93.02%	1.837%	96.45%	2.141%	95.15%
Best	0.740%	99.39%	0.596%	99.68%	0.719%	99.41%	0.647%	99.78%
Worst	2.636%	92.96%	3.779%	89.81%	2.731%	77.36%	2.563%	95.15%
Average	1.753%	96.12%	2.113%	95.72%	1.493%	94.77%	1.622%	97.46%

**Figure A1:**
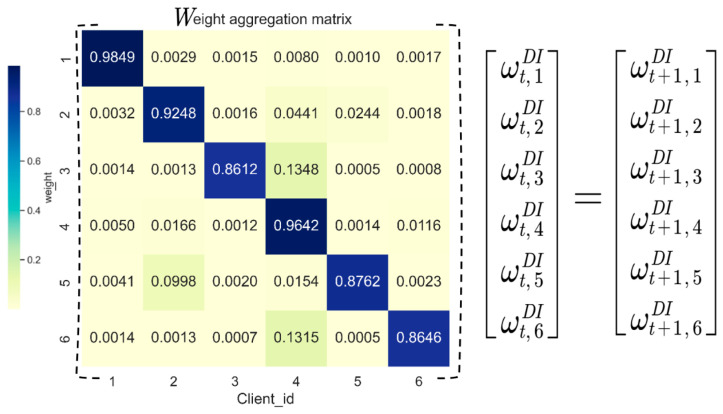
Visual example of the personalized parameter aggregation process.

## Data Availability

The data presented in this study are publicly available in the SDUMLA-HMT, MMCBNU-6000, HKPU-FV, NUPT-FV, VERA, and UTFVP datasets [21,31,33,34,35,36].

## Appendix A. Proof of Convergence

Based on the L-Lipschitz smoothness of the *f* Taylor expansion, we can obtain the following inequality, as shown in Equation (A1).

(A1) $$f\left({\bar{w}}_{t+1}^{DI}\right)\leq f\left(w_{t}^{DI}\right)+<\nabla f\left(w_{t}^{DI}\right),{\bar{w}}_{t+1}^{DI}-w_{t}^{DI}>+\frac{L}{2}\|{\bar{w}}_{t+1}^{DI}-w_{t}^{DI}\|$$

(A2) $$E_{St}\left[<\nabla f\left(w_{t}^{DI}\right),{\bar{w}}_{t+1}^{DI}-w_{t}^{DI}>\right]=E_{St}\left[<\nabla f\left(w_{t}^{DI}\right),E_{k}\left[w_{t+1,i}^{DI}-w_{t}^{DI}\right]>\right]\;=-\eta E_{St}\left[<\nabla f\left(w_{t}^{DI}\right),E_{k}\left[\nabla L_{k}\left(w_{k}^{DI},w_{k}^{DS},w_{k}^{Dec},\varphi_{k}\right)\right]>\right]$$

Additionally, for vectors ***a*** and ***b***, we have Equation (A3).

(A3) $$<a,b>=\frac{1}{2}\left(\|a{\|}^{2}+\|b{\|}^{2}-\|a-b{\|}^{2}\right)$$

Below, we obtain the bounds for $E_{St}\left[<\nabla f\left(w_{t}^{DI}\right),{\bar{w}}_{t+1}^{DI}-w_{t}^{DI}>\right]$ and $\left\|{\bar{w}}_{t+1}^{DI}-w_{t}^{DI}\right\|$ via mathematical deflation. Equations (A3), (A2) and (10) can be combined, yielding Equation (A4).

(A4) $$E_{St}\left[<\nabla f\left(w_{t}^{DI}\right),{\bar{w}}_{t+1}^{DI}-w_{t}^{DI}>\right]\;\leq -\frac{\eta }{2}E_{St}\left(\|\nabla f\left(w_{t}^{DI}\right){\|}^{2}+\|\nabla L_{k}\left(w_{t,k}^{DI},w_{t,k}^{DS},w_{t,k}^{Dec},\varphi_{t,k}\right){\|}^{2}-\|\nabla f\left(w_{t}^{DI}\right)-\nabla L_{k}\left(w_{t,k}^{DI},w_{t,k}^{DS},w_{t,k}^{Dec},\varphi_{t,k}\right){\|}^{2}\right)\;\leq -\frac{\eta }{2}E_{St}\left(\|\nabla f\left(w_{t}^{DI}\right){\|}^{2}+\|\nabla L_{k}\left(w_{t,k}^{DI},w_{t,k}^{DS},w_{t,k}^{Dec},\varphi_{t,k}\right){\|}^{2}\right)$$

Equation (A5) can be obtained by combining Equations (12) and (A4).

(A5) $$E_{St}\left[<\nabla f\left(w_{t}^{DI}\right),{\bar{w}}_{t+1}^{DI}-w_{t}^{DI}>\right]\leq -\frac{\eta }{2}\left(B^{2}+1\right)\|\nabla f\left(w_{t}^{DI}\right){\|}^{2}$$

The upper bound for $\left\|{\bar{w}}_{t+1}^{DI}-w_{t}^{DI}\right\|$ is solved as follows: Let ${\hat{w}}_{t+1,k}^{DI}=arg\min_{w^{DI}}L_{k}\left(w_{t,k}^{DI},w_{t,k}^{DS},w_{t,k}^{Dec},\varphi_{t,k}\right)$. Combining Equations (9) and (11), we obtain Equations (A6) and (A7).

(A6) $$\left\|{\hat{w}}_{t+1,k}^{DI}-w_{t+1,k}^{DI}\|\leq \frac{\gamma }{\mu }\|\nabla L_{k}\left(w_{t,k}^{DI},w_{t+1,k}^{DS},w_{t+1,k}^{Dec},\varphi_{t+1,k}\right)\right\|$$

(A7) $$\left\|{\hat{w}}_{t+1,k}^{DI}-w_{t}^{DI}\|\leq \frac{1}{\mu }\|\nabla L_{k}\left(w_{t,k}^{DI},w_{t+1,k}^{DS},w_{t+1,k}^{Dec},\varphi_{t+1,k}\right)\right\|$$

Additionally, for vectors ***a*** and ***b***, we have the triangular inequality shown in Equation (A8).

(A8) ‖a−b‖≤‖a−c‖+‖b−c‖

Equation (A9) can be obtained by combining Equations (A6) and (A7).

(A9) $$\left\|w_{t+1,k}^{DI}-w_{t}^{DI}\|\leq \|{\hat{w}}_{t+1,k}^{DI}-w_{t+1,k}^{DI}\|+\|{\hat{w}}_{t+1,k}^{DI}-w_{t}^{DI}\right\|\;=\frac{\gamma +1}{\mu }\|\nabla L_{k}\left(w_{t+1,k}^{DI},w_{t+1,k}^{DS},w_{t+1,k}^{Dec},\varphi_{t+1,k}\right)\|$$

Therefore, we can deduce Equation (A10).

(A10) $$\|{\bar{w}}_{t+1}^{DI}-w_{t}^{DI}\|\leq E_{k}\left[\left\|w_{t+1,k}^{DI}-w_{t}^{DI}\right\|\right]=\frac{\gamma +1}{\mu }\sqrt{E_{k}\left[\|\nabla L_{k}\left(w_{t,k}^{DI},w_{t,k}^{DS},w_{t,k}^{Dec},\varphi_{t,k}\right){\|}^{2}\right]}\;\leq \frac{B\left(1+\gamma \right)}{\mu }\|\nabla f\left(w_{t}^{DI}\right){\|}^{2}$$

Combining Equations (A1), (A5) and (A10) leads to Equation (A11).

(A11) $$f\left({\bar{w}}_{t+1}^{DI}\right)\leq f\left(w_{t}^{DI}\right)+<\nabla f\left(w_{t}^{DI}\right),{\bar{w}}_{t+1}^{DI}-w_{t}^{DI}>+\frac{L}{2}\|{\bar{w}}_{t+1}^{DI}-w_{t}^{DI}\|\;\leq f\left(w_{t}^{DI}\right)+\left(\frac{LB(1+\gamma )}{2\mu }-\frac{\eta }{2}\left(B^{2}+1\right)\right)\|\nabla f\left(w_{t}^{DI}\right){\|}^{2}$$

Next, the local Lipschitz smoothness of *f* is utilized to find the relationship between $E\left[f\left(w_{t+1}^{DI}\right)\right]$ and $f\left(w_{t}^{DI}\right)$ to obtain Equation (A12).

(A12) $$f\left(w_{t+1}^{DI}\right)\leq f\left({\bar{w}}_{t+1}^{DI}\right)+L_{0}\|{\bar{w}}_{t+1}^{DI}-w_{t+1}^{DI}\|$$

where *L*_0_ is the local Lipschitz continuity constant for function *f* and is formulated as shown in (A13).

(A13) $$L_{0}\leq \|\nabla f\left(w_{t}^{DI}\right)\|+L\cdot \max\left(\left\|{\bar{w}}_{t+1}^{DI}-w_{t}^{DI}\|,\|w_{t+1}^{DI}-w_{t}^{DI}\right\|\right)\;\leq \|\nabla f\left(w_{t}^{DI}\right)\|+L\left(\left\|{\bar{w}}_{t+1}^{DI}-w_{t}^{DI}\|+\|w_{t+1}^{DI}-w_{t}^{DI}\right\|\right)$$

Therefore, to obtain the calculated expectation value of the selected device in round *t*, we need to define Equation (A14).

(A14) $$E_{St}\left[f\left(w_{t+1}^{DI}\right)\right]\leq f\left({\bar{w}}_{t+1}^{DI}\right)+O_{t}$$

where $O_{t}$ satisfies the inequality shown in (A15).

(A15) $$O_{t}\leq E_{St}\left[\left\|\nabla f\left(w_{t}^{DI}\right)\|+L\left(\left\|{\bar{w}}_{t+1}^{DI}-w_{t}^{DI}\|+\|w_{t+1}^{DI}-w_{t}^{DI}\right\|\right)\|w_{t+1}^{DI}-{\bar{w}}_{t+1}^{DI}\right\|\right]\;\leq \left(\left\|\nabla f\left(w_{t}^{DI}\right)\|+2L\|{\bar{w}}_{t+1}^{DI}-w_{t}^{DI}\right\|\right)E_{St}\left[\left\|w_{t+1}^{DI}-{\bar{w}}_{t+1}^{DI}\right\|\right]+LE_{St}\left[\|w_{t+1}^{DI}-{\bar{w}}_{t+1}^{DI}{\|}^{2}\right]$$

The following process for $E_{St}\left[\|w_{t+1}^{DI}-{\bar{w}}_{t+1}^{DI}{\|}^{2}\right]$ and $E_{St}\left[\left\|w_{t+1}^{DI}-{\bar{w}}_{t+1}^{DI}\right\|\right]$ is performed based on the inequality in (A16).

(A16) $$E_{St}\left[\left\|w_{t+1}^{DI}-{\bar{w}}_{t+1}^{DI}\right\|\right]\leq \sqrt{E_{St}\left[\|w_{t+1}^{DI}-{\bar{w}}_{t+1}^{DI}{\|}^{2}\right]}$$

$$\begin{array}{c} E_{St}\left[{∥w_{t+1}^{DI}-{\bar{w}}_{t+1}^{DI}∥}^{2}\right]\leq E_{St}\left[{∥\frac{1}{K}\underset{k\in St}{\Sigma }\;w_{t+1,k}^{DI}-{\bar{w}}_{t+1}^{DI}∥}^{2}\right]\\ \leq \frac{1}{K^{2}}E_{St}\left[{∥\underset{k\in St}{\Sigma }\;w_{t+1,k}^{DI}-K{\bar{w}}_{t+1}^{DI}∥}^{2}\right]\\ \leq \frac{1}{K^{2}}E_{St}\left[\underset{k\in St}{\Sigma }\;{∥w_{t+1,k}^{DI}-{\bar{w}}_{t+1}^{DI}∥}^{2}\right]\\ =\frac{1}{K}E_{k}\left[{∥w_{t+1}^{DI}-{\bar{w}}_{t+1}^{DI}∥}^{2}\right]\\ =\frac{1}{K}E_{k}\left[{∥w_{t+1}^{DI}-E_{k}\left[w_{t+1,k}^{DI}\right]∥}^{2}\right]\\ \leq \frac{1}{K}\left(E_{k}\left[{∥w_{t+1,k}^{DI}-w_{t}^{DI}∥}^{2}\right]+E_{k}\left[{∥E_{k}\left[w_{t+1,k}^{DI}\right]-w_{t}^{DI}∥}^{2}\right]\right)\\ \leq \frac{2}{K}E_{k}\left[{∥w_{t+1,k}^{DI}-w_{t}^{DI}∥}^{2}\right] \end{array}$$

Considering Equation (A9), we obtain the inequality shown in (A17).

(A17) $$E_{St}\left[{∥w_{t+1}^{DI}-{\bar{w}}_{t+1}^{DI}∥}^{2}\right]\leq \frac{B^{2}(1+\gamma {)}^{2}}{K\mu^{2}}{∥\nabla f\left(w_{t}^{DI}\right)∥}^{2}$$

The inequality in (A18) is obtained by substituting Equations (A10), (A16) and (A17) into Equation (A15).

(A18) $$O_{t}\leq \left(\frac{\sqrt{2}B(1+\gamma )}{\mu \sqrt{K}}+(2\sqrt{2}+2)\frac{B^{2}L{\left(1+\gamma \right)}^{2}}{\mu^{2}K}\right)\|\nabla f\left(w_{t}^{DI}\right){\|}^{2}$$

Combining Equations (A11), (A14) and (A18) leads to Equation (A19).

(A19) $$E_{St}\left[f\left(w_{t+1}^{DI}\right)\right]\leq f\left(w_{t}^{DI}\right)-\;\left[\frac{\eta }{2}\left(B^{2}+1\right)-\frac{BL\left(1+\gamma \right)}{2\mu }-\left(2\sqrt{2}+2\right)\frac{B^{2}L{\left(1+\gamma \right)}^{2}}{\mu^{2}K}-\frac{\sqrt{2}B\left(1+\gamma \right)}{\mu \sqrt{K}}\right]\|\nabla f\left(w_{t}^{DI}\right){\|}^{2}$$

Then, for the chosen learning rate *η*, the total number of participating clients *K*, *γ*, and *μ* are selected to satisfy Equation (A20).

(A20) $$\alpha =\left(\frac{\eta }{2}(B^{2}+1)-\frac{BL(1+\gamma )}{2\mu }-(2\sqrt{2}+2)\frac{B^{2}L{\left(1+\gamma \right)}^{2}}{\mu K}-\frac{\sqrt{2}B(1+\gamma )}{\mu \sqrt{K}}\right)>0$$

Then, the expected decline in the global target in round *t* is shown as the inequality in (A21).

(A21) $$E_{St}\left[f\left(w_{t+1}^{DI}\right)\right]\leq f\left(w_{t}^{DI}\right)-\alpha \|\nabla f\left(w_{t}^{DI}\right){\|}^{2}$$

We obtain the inequality in (A22) by summing the inequalities for the *T* round iterations of the operation shown in (A21).

(A22) $$f^{*}-f\left(w_{0}^{DI}\right)\leq -\alpha \underset{t=0}{\overset{t-1}{\Sigma }}\|\nabla f\left(w_{t}^{DI}\right){\|}^{2}$$

Therefore, the converged form is finally derived as shown in Equation (A23).

(A23) $$\frac{1}{T}\overset{t-1}{\underset{t=0}{\Sigma }}\;{∥\nabla f\left(w_{t}^{DI}\right)∥}^{2}\leq -\frac{f\left(w_{0}^{DI}\right)-f^{*}}{\alpha T}$$

This equation shows that as the number of iterations *T* increases, the upper bound on this average gradient paradigm decreases, eventually converging to a stable value.

## Appendix B. The Aggregation Weight Matrix in the Experiments

As shown in Figure A1, this weight matrix corresponds to the six clients, SDU, MMC, HKPU, NUPT, VERA, and UTFVP, in order from top to bottom. *w*[*k*][*z*] denotes the proportion of the model parameters uploaded by the *z*-th client when calculating the parameters of the *k*-th client. The degree of dependence of each client on the information of other clients is not the same, taking the SDU in the first row as an example. To aggregate a model that is more suitable for the distribution of the SDU dataset, the locally trained model parameters account for a large portion of a small amount of access to the knowledge of the other clients, and for the VERA client, the other clients must provide more information to aggregate a model that performs better based on the VERA data.
